# Translational and Comparative Research on Innovative Anti-Cancer Therapies

**DOI:** 10.3390/cancers15041335

**Published:** 2023-02-20

**Authors:** Felisbina Queiroga, Bruno Cogliati

**Affiliations:** 1 Department of Veterinary Sciences, University of Trás-os-Montes and Alto Douro, 5000-801 Vila Real, Portugal; 2 Animal and Veterinary Research Centre (CECAV), University of Trás-os-Montes e Alto Douro, 5000-801 Vila Real, Portugal; 3 Center for the Study of Animal Sciences, CECA-ICETA, University of Porto, 4200-465 Porto, Portugal; 4 Department of Pathology, School of Veterinary Medicine and Animal Science, University of São Paulo, Av. Prof. Dr. Orlando Marques de Paiva 87, 05508-270 São Paulo, Brazil

Oncology research has received considerable attention in recent years due to the increasing prevalence of cancer in human and animal populations worldwide. The similarities between neoplastic diseases that affect both species have been identified in terms of etiopathogenesis, serum and immunohistochemical biomarkers, and gene expression, as well as their response to antineoplastic drugs, for example. Therefore, several translational and comparative studies are described in the literature, placing cancer in a one health perspective and simultaneously improving the anti-cancer therapies provided in human and veterinary oncology. In this Special Issue, eleven studies with relevant scientific information regarding innovative anti-cancer therapies were included in the context of comparative oncology.

This Special Issue starts with an original study in which the influence of folic acid was investigated in the genetic and epigenetic regulation of two colorectal cancer cell lines (HT-29 and SW480) [1]. Zsigrai et al. [1] showed that the short-term folic acid supplementation affected HT-29 cell proliferation, viability, and genomic stability, although similar findings were not found in the SW480 cell line. Moreover, gene downregulation and upregulation were identified in both cell lines, contributing to new information about the effect of this vitamin in different human colorectal cancer cell lines. Moving from a nutritional supplement to a cytotoxic drug, Ferrari et al. [2] first described the use of Bleosome, a new compound consisting of ultra-deformable liposomes with encapsulated bleomycin for topical administration, evaluating its ability to penetrate ex vivo skin explants from dogs and horses. The authors showed that Bleosome was able to penetrate the skin and release bleomycin into deeper epidermal layers, which could represent a more effective and safer anti-cancer therapy for non-melanoma skin cancer compared to the conventional systemic treatments.

In the context of comparative oncology, canine mammary gland neoplasia is commonly used as a translational study model for breast cancer in women due to its complex nature and biological behavior. In order to find potential prognostic biomarkers, a proteomic mass spectrometry imaging technique was performed by Cordeiro et al. [3] in mammary tumors surgically removed from dogs diagnosed with metastatic neoplastic disease. The authors associated the malignant tumor phenotype with alterations in the expression of five proteins: Fibronectin type III domain containing 1 protein (FNDC1), Alpha-1B-glycoprotein (A1BG), calnexin (CANX), heat-shock protein family A member 5 (HSPA5), and protein disulfide isomerase family A member 3 (PDIA3). Based on these findings, it was suggested that these five key proteins could be potential prognostic biomarkers for this type of cancer, but further investigation and validation are still required. In turn, Colombo et al. [4] showed the effectiveness of liquid biopsy as a diagnostic and prognostic tool for both women and female dogs diagnosed with breast cancer through the identification of several specific genetic mutations in the respective neoplastic fragments.

Head and neck squamous cell carcinoma (HNSCC) was also addressed in this Special Issue as Da Silva et al. [5] described a novel matriptase-dependent proteolytic pathway associated with the activation of the protease-activated receptor 3 (PAR-2) by the Kallikrein 5 (KLK5), which represents a modulatory mechanism of potential clinical interest in the carcinogenesis of this cell type. Moreover, as the authors identified an inhibitory action of the serine protease inhibitor lymphoepithelial Kazal-type-related inhibitor (LEKTI) on KLK5, it is expected that these results motivate the investigation of new and related targeted therapies in these tumors. Still focusing on HNSCC, Piotrowski et al. [6] added relevant diagnostic information, describing the potential clinical interest of microRNAs expression in differentiating between tumor and healthy tissues. In addition, the authors reported an association between this expression and the patient outcome, suggesting the potential use of these biomarkers as predictors of survival.

Regarding innovative anti-cancer therapies, Kung et al. [7] showed the anti-cancer effect of piperlongumine, an amide alkaloid, in human thyroid cancer mainly due to its ability to inhibit cell proliferation and to promote apoptosis by generating reactive oxygen species. As this study was performed in vitro and in vivo, its potential applicability as an effective and safe anti-cancer therapy in humans is very promising. In dogs, Voges et al. [8] reported the effectiveness of weekly intratumoral doses of polyinosinic-polycytidylic acid-poly-l-lysine carboxymethylcellulose (poly-ICLC) in a clinical study with advanced and unresectable canine tumors for the first time. This anticancer therapy improved the qualities of life of these dogs and was considered locally effective and well tolerated, with only mild adverse effects being described.

At the end, the readers will find three comprehensive review papers that contribute up-to-date evidence on different topics associated with translational and comparative oncology research. In the first one, Torres-Juárez et al. [9] addressed the neurobiology of cancer, the impact of the nervous system in carcinogenesis events, and the influence of this interaction on anti-cancer approaches. Secondly, Romualdo et al. [10] described the main in vivo and in vitro models of hepatocellular carcinoma, regarding their respective advantages, disadvantages, and applications. Finally, Wawruszak et al. [11] explored the impact of the single or combined use of valproic acid, a short-chain fatty acid, in the treatment of breast cancer, through the information available in the peer-reviewed literature.

In conclusion, we believe that this Special Issue of *Cancers* contributes with high-quality scientific evidence through novel data from preclinical and clinical studies performed in humans and/or animals. Thus, we hope that this set of articles will increase readers’ knowledge within the scope of comparative and translational oncology and that it will encourage further research into these innovative anti-cancer therapies.
